# Fabrication of Mildew-Resistant Wood with Multi-Functional Properties Based on In Situ Growth of Metal–Organic Frameworks

**DOI:** 10.3390/polym16030313

**Published:** 2024-01-23

**Authors:** Xingyu Liang, Tao Zhang, Junting Li, Wei Wang, Tiancheng Yuan, Yanjun Li

**Affiliations:** 1 Jiangsu Co-Innovation Center of Efficient Processing and Utilization of Forest Resources, Nanjing Forestry University, Nanjing 210037, China; 2 Bamboo Research Institute, Zhejiang Agriculture and Forestry University, Hangzhou 311300, China

**Keywords:** wood, MOFs, superhydrophobic property, self-cleaning property, anti-mildew property

## Abstract

Wood is easily affected by decay fungi, mildew fungi, insects, water, UV, and other factors when used outdoors. In particular, mildew on the surface of wood negatively affects the appearance and practical use of wood or wood-based engineered products. In recent years, as a class of popular crystalline materials, metal–organic frameworks (MOFs) have been widely applied in electrochemistry, adsorption, anti-mildew efforts, and other areas. In this study, we first grew a Co-based metal–organic framework (Co-MOF) in situ on a wood surface and subsequently converted the Co-MOF in situ into a cobalt–nickel double hydroxide layer, which formed micro- and nanohierarchical composite structures on the wood surface. The low surface energy of the CoNi-DH@wood was further modified via impregnation with sodium laurate to obtain the superhydrophobic wood (CoNi-DH-La@wood). We characterized the microstructure, chemical composition, water contact angle, and anti-mold properties of the CoNi-DH-La@wood using SEM, XRD, XPS, water contact angle tests, and anti-fungal tests. The SEM, XRD, and XPS results confirmed that the metal–organic framework was coated on the wood surface, with the long-chain sodium laurate grafted onto it. The CoNi-DH-La@wood had a water contact angle of 151°, demonstrating excellent self-cleaning ability. In addition, the fabricated superhydrophobic balsa wood exhibited excellent chemical and environment stability. Lastly, the CoNi-DH-La@wood exhibited excellent anti-mildew properties in a 30-day anti-mildew test because the superhydrophobic coating was successfully coated on the wood surface. In summary, this work presents an attractive strategy for obtaining wood with superhydrophobic properties at room temperature, thereby endowing the wood or wood-based engineered products with excellent anti-mildew properties.

## 1. Introduction

For the last few years, bio-inspired superhydrophobic surfaces have attracted more attention in industrial and research applications [1]. As a green, renewable, inexpensive, easily harvested, biodegradable, and abundant resource, balsa wood has great potential for the development of multi-functional composite materials because of its unique chemical components and natural hierarchical porous structure [2], which can be achieved by exploiting the unique chemical components, high porosity, and natural hierarchical structure of wood in one step. In past decades, wood has been functionalized as a template with multiple functions and applications, such as for lightweight structural materials, transparent windows, oil–water separation, water processing, solar evaporation, and other functions. Unfortunately, biomass materials such as wood are easily affected by decay fungi, mildew fungi, insects, water, UV, and other factors when applied in outdoor environments [3,4,5]. In particular, mildew on the surface of wood negatively affects the appearance and practical use of wood and wood-based engineered products [6]. At present, chemical modification and physical modification are the two main solutions for preventing wood mildew, and the widely applied operations include thermal modification, soaking, and dipping methods. The traditional processes usually involve direct immersion of wood or wood-based engineered products in an anti-fungal chemical agent to solve the problem of molding [7]. However, this direct immersion strategy not only requires a large amount of wasted chemical reagents and a poor loading rate but also results in harmful chemical residues on the surface of the wood or wood-based engineered product, which can negatively affect the ecosystem, human health, or animal health in daily applications [8]. Thus, it is of great importance to develop a green, simple, and environmentally friendly method that can harness the excellent anti-fungal properties of wood and wood-based engineering materials.

Bionic, artificial superhydrophobic coatings can be formed by combining low surface free energy components with hierarchical nano- or microstructures [9]. Furthermore, these fabricated superhydrophobic coatings endow the wood or wood-based product with multiple functions such as self-cleaning, anti-fogging, anti-corrosion, drag reduction, mildew resistance, and other functions. In other words, the fabricated superhydrophobic coating forces the surface to have a small water contact angle and a tiny water–solid contact area, endowing the wood surface with advanced functions [10,11,12]. Interestingly, nutrients and water are two important factors for the growth of *Aspergillus niger*. The successful construction of a superhydrophobic coating on a wood surface can effectively prevent the exchange of nutrients between the wood and the outside environment, and the water-repellent capacity of the wood surface can effectively prevent the growth of *Aspergillus niger* [13,14,15].

In recent years, as a class of popular crystalline materials, metal–organic frameworks (MOFs) have been widely applied in electrochemistry, adsorption, anti-mildew, and catalyst functions [16,17,18,19]. Additionally, as promising solid matrixes, MOFs have gained more attention owing to their diverse advantages, including their tunable pore size, high stability, and large specific surface area [19]. Co-based metal–organic frameworks (Co-MOFs) present excellent thermal stability, involve a simple manufacturing process, and have good chemical stability, meaning they can be easily fabricated on wood surfaces as hierarchical nano- or microstructures [20,21,22]. Thus, the combination of MOFs and low surface energy substances to give biomass materials a variety of functions has attracted the interest of researchers and has been a hot topic in recent years. Balsa wood has received tremendous attention due to its ease of harvest, low density, low cost, and excellent mechanical properties [23,24]. Wood has a unique microporous structure that can be used as a load-bearing template for MOFs, providing a large number of active sites conducive to the loading of MOFs and enabling the development and application of wood-based advanced materials [25,26,27]. Additionally, wood mainly consists of hemicellulose, cellulose, and lignin. As we known, the hemicellulose and cellulose molecules contain carboxyl (-COOH) groups and hydroxyl (-OH) groups, which can help in efficiently loading MOFs on wood surfaces. In recent years, there have been numerous reports of the use of wood-based materials for electromagnetic wave absorption, electromagnetic shielding, energy storage, desalination, and other applications [28,29,30]. Wei et al. prepared a highly anisotropic MXene@wood composite with convenient shielding effectiveness (SE) tuning by coating MXene on natural wood surfaces [31]. Zhang et al. rationally designed a bilayered evaporator consisting of a black Chinese ink layer and wood sheet substrate for continuous desalination [32]. However, the reports focusing on the use of wood and wood-based engineering materials for surface property enhancements and mold resistance are very limited. The preparation of green, environmentally friendly functional woods by combining MOFs to improve the service life of wood-based engineering materials is a very important and urgent issue to be addressed.

In this work, a multi-functional and superhydrophobic coating was successfully fabricated on balsa wood surfaces through a simple method involving the in situ growth of metal–organic frameworks. The micromorphology, chemical compositions, chemical stability, durability under harsh condition, water contact angle, and other aspects were characterized using scanning electron microscopy (SEM), X-ray photoelectron spectrometry (XPS), X-ray diffraction (XRD), and mechanical tests. In addition, the anti-mildew performance of the superhydrophobic wood was also assessed. In this paper, we propose a method for the fabrication of superhydrophobic, mold-resistant wood via the in situ growth and transformation of MOFs at room temperature. This method is simple, does not require complex reaction conditions, does not require wasted energy, and is viable for future applications in the development of wood-based functional materials, as well as providing a new idea for wood processing companies and researchers in biomass materials.

## 2. Materials and Methods

### 2.1. Materials

The balsa wood was manufactured into 50 × 50 × 2 mm (length × width × thickness) samples. Nickel sulfate heptahydrate, 2-methylimidazole (2-MIN), cobalt(II) nitrate hexahydrate (Co(NO_3_)_2_. 6H_2_O), and hydrogen peroxide (H_2_O_2_) were purchased from Nanjing Banma Industry Co., Ltd. (Nanjing, China). In addition, sodium laurate (La, 98%) was purchased from Aladdin Reagent Company (Shanghai, China).

### 2.2. Fabrication Process of Superhydrophobic Surfaces on Balsa Wood

**Scheme 1:**
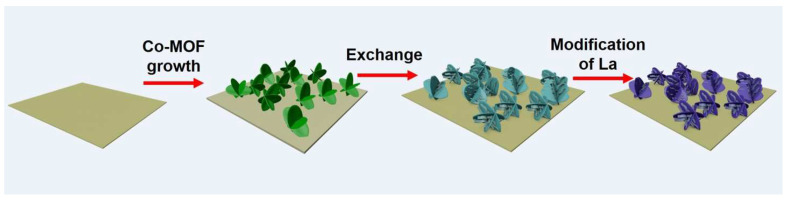
Fabrication process and structural design of CoNi-DH-La@Wood.

**Figure 1:**
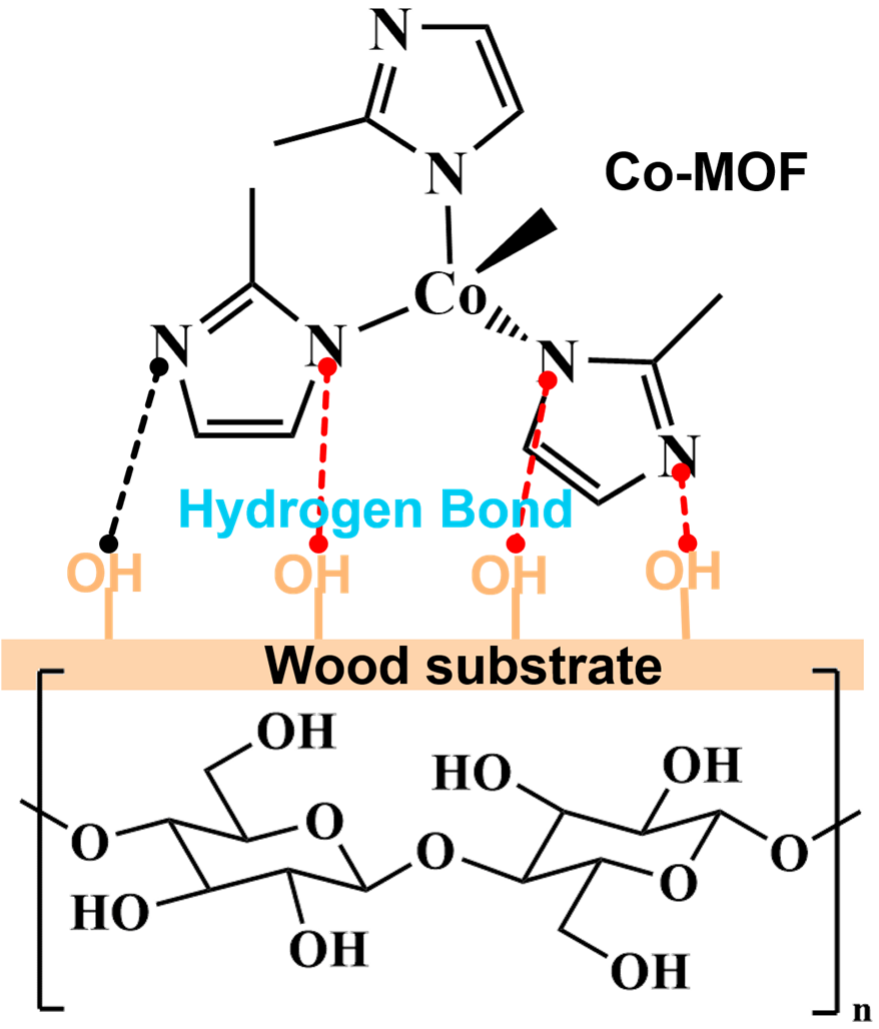
Reaction mechanism of superhydrophobic wood surfaces.

Firstly, the sliced balsa wood samples were cut into an average size of 50 × 50 × 2 mm (length × width × thickness) and then the wood surface was sanded with 500-grit emery sandpaper to remove the surface stains, thereby obtaining flat and smooth wood surfaces. Then, the sanded sliced balsa wood samples were soaked in a mixed solution of 0.05 M of (Co(NO_3_)_2_. 6H_2_O) and 0.4 M of 2-MIN and left for 8 h at room temperature, enabling the in situ growth of the Co-based metal–organic framework (Co-MOF) on the balsa wood surface. Then, one obtained wood sample was washed using deionized water and ethanol and dried in an oven at 103 °C for 12 h. This wood sample was named Co-MOF@Wood. Thirdly, the Co-MOF@Wood specimen was soaked in 16 mM of NiSO_4_·7H_2_O for 10 h at room temperature to accomplish the in situ conversion of the Co-MOF. Then, the obtained wood sample was washed with deionized water and ethanol and dried in an oven at 103 °C for 10 h. This wood sample was named CoNi-DH@Wood. Lastly, the CoNi-DH@Wood was soaked in a 0.05 M sodium laurate (La) solution for 10 h, then the CoNi-DH-La@Wood was fished out of the 0.05 M sodium laurate (La) solution with tweezers and air-dried in a natural environment. For detail, the natural drying process can effectively reduce the wood surface’s energy, endowing the wood samples with superhydrophobic performance. This wood sample was labeled CoNi-DH-La@Wood. The flowchart for the preparation of the superhydrophobic wood samples is shown in Scheme 1. The reaction mechanism of the superhydrophobic wood surfaces is presented in Figure 1.

### 2.3. Characterization

The chemical compositions of the different wood samples were characterized using X-ray photoelectron spectroscopy (XPS, DLD, Munich, Germany). Additionally, the micromorphologies of the sample surfaces were observed via scanning electron microscopy (SEM, Quanta 3000, Tokyo, Japan). In addition, the superhydrophobic samples were individually immersed in organic solutions of different pH values for 48 h and then dried at 103 °C for 12 h, and the change in water contact angle of the superhydrophobic wood blocks after immersion was measured to determine the chemical durability of the superhydrophobic layers. The water contact angle of the superhydrophobic wood blocks was measured after immersion in water for 60 min and drying at 103 °C for 12 h to evaluate the resistance of the superhydrophobic coating to ultrasonic cleaning. Finally, the water contact angle of the superhydrophobic wood blocks was measured after being exposed to UV light for 12 h to evaluate the UV resistance of the superhydrophobic coating. The power of the UV light was 40 w and the radiation wavelength of the UV light was 365 nm. The contact angle (CA) and sliding angle (SA) of the sample surfaces were determined on an optical contact angle-measuring device (Dataphysics, Filderstadt, Germany) at room temperature with a droplet volume of 5 μL.

### 2.4. Anti-Mildew Test

Firstly, 46 g of potato dextrose agar (PDA) powder was weighed and set aside. Then, a beaker with a capacity of 2000 mL was connected to 1000 mL of deionized water and the 46 g of PDA powder weighed in the previous step was introduced into the deionized water. The mixture was placed on a magnetic stirrer and stirred at 900 rpm/min for 45 min until the PDA powder was completely dissolved in the deionized water. Three round-necked glass bottles with a capacity of 3000 mL were then taken, the PDA mixture was poured into each of the three round-necked glass bottles, and the bottles were wrapped in plastic wrap. Afterwards, the applicators, catcher sticks, petri dishes, and U-rods needed for the subsequent tests were placed in an autoclave bag. Both the above solutions and the required glass apparatus were placed in a sterilizing pot and sterilized at 121 °C for 30 min. After sterilization, the sterilized test items were transferred to an ultra-clean bench for UV sterilization for 30 min. After UV sterilization, the sterilized PDA solution was transferred to a Petri dish and then UV-sterilized for 50 min and left to set. Finally, the prepared *Aspergillus niger* was evenly inoculated on the PDA gel using an inoculating rod and applicator. All Petri dishes were sealed with sealing strips and transferred to a constant temperature and humidity chamber, where they were incubated at 25 °C and 80% humidity for one week to await mycelial development.

**Figure 2:**
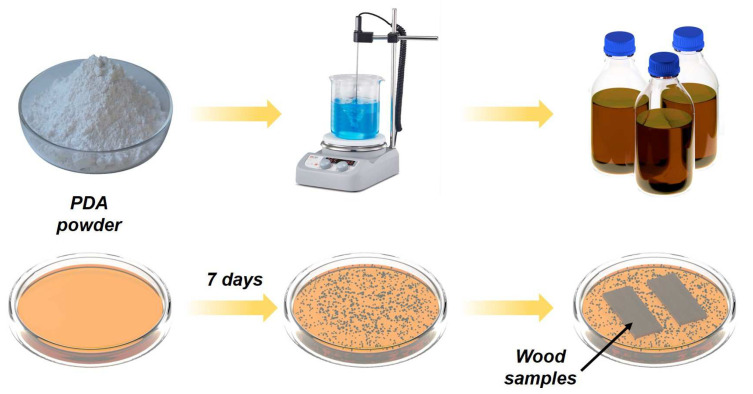
A schematic diagram of the anti-mildew test process.

After one week, the samples to be tested were first placed in an autoclave bag and tied with a rubber band and then placed in a sterilizing pan at 121 °C for 30 min. In this case, the temperature of the sterilizer was raised at 5 °C/min and lowered at 5 °C/min. Afterwards, the samples were transferred to an ultra-clean bench together with the inoculated molds from the previous step for UV sterilization for 30 min. Finally, the samples were inoculated on a culture medium and incubated at 25 °C constant temperature and 80% constant humidity for one month, and the mold growth on the surfaces of the samples was recorded using digital camera for the observation and recording of test data. A schematic diagram of the anti-mildew test process is presented in Figure 2.

## 3. Results

### 3.1. Microstructure Characterization

**Figure 3:**
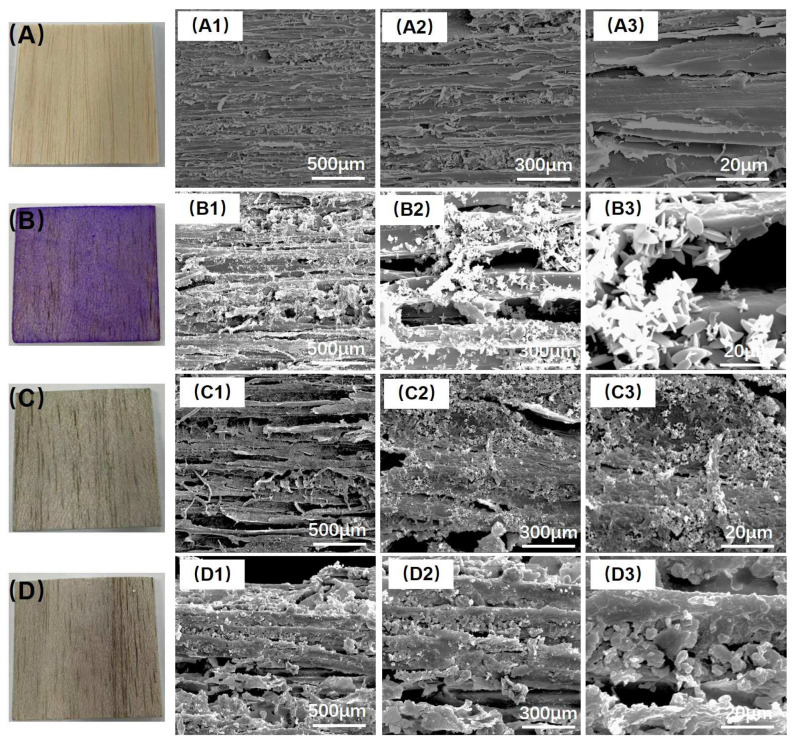
Physical photographs of the different wood samples and corresponding SEM images: (**A**–**A3**) balsa wood; (**B**–**B3**) Co-MOF@wood; (**C**–**C3**) CoNi-DH@wood; (**D**–**D3**) CoNi-DH-La@wood.

To begin with, the schematic diagram for fabricating superhydrophobic coatings on the balsa wood surface was analyzed further. Firstly, we introduced the 2-MIN into 0.05 M of Co(NO_3_)_2_. In the 6H_2_O solution, the Co-MOF grew in situ on the balsa wood surface at room temperature. Secondly, the Co-MOF was converted to CoNi-DH on the surface of the balsa wood by adding the appropriate amount of NiSO_4_·7H_2_O solution, mainly through an exchange process between the organic ligands of the Co-MOF and the hydroxide ions during the hydrolysis process. In Figure 3B(1)–B(3), it can been seen from the SEM images that the Co-MOF completely and uniformly covered the surface of the balsa wood, and the physical photograph of the Co-MOF@wood exhibits a purple color, as confirmed in Figure 3. However, the purple color faded from the surface of the obtained CoNi-DH@wood due to the presence of Ni^2+^ and SO_4_^2−^. Further, after coating the surface with sodium laurate, the surface of the CoNi-DH-La@wood was faintly white, and the SEM results showed that the surface of the wood was intact and presented a micro- or nano-hierarchical structure on wood surface. The benefits of the method used for constructing micro- and nanohierarchical structures via the in situ growth and in situ transformation of MOFs, which are then further combined with low surface energy substances to impart functionality to the wood, are three-fold: (1) the entire experimental process is simple, as no strong chemicals are used; (2) no high temperatures are involved in the reaction and the experiment is safe; (3) this method for the in situ growth and in situ transformation of MOFs and further combination of low surface energy substances does not damage the surface or internal cell walls of the wood and is suitable for industrial production.

Scanning electron microscopy (SEM) was further applied to observe the micromorphologies and microstructures of different wood samples, as presented in Figure 3. Expectedly, the Co-MOF precursors exhibit a sheet-like structure and are clustered on the wood’s cell wall and inside the wood’s cell lumen. With the introduction of the NiSO_4_·7H_2_O solution, the Co-MOF is converted into CoNi-DH at room temperature, and the CoNi-DH exhibits a three-dimensional hierarchical structure with CoNi-DH interconnected and uniformly distributed on the wood surface, forming a micro- or nano-hierarchical structure on the wood surface. After being further modified with La, it can been seen from Figure 3D that the surface of the obtained CoNi-DH-La nanosheets became rougher and the nanosheets on wood surface changed from a sheet-like structure to irregular particles. This can be attributed to the hydrated interlayer galleries of CoNi-DH that were inserted by La ions via the ion exchange process [23,33,34]. Considerable stress is generated during this exchange process, and as a result the structure of the hydroxide changes dramatically and a rearrangement between the molecules occurs to cope with and release this stress. Fortunately, a micro- and nanohierarchical composite structures were successfully fabricated on the wood surface, and the surface energy of the wood was decreased by the sodium laurate’s long organic chains. The chemical components, macromechanical properties, durability, stability, and mildew resistance of different wood specimens will be further explored in this work.

### 3.2. XRD and XPS Analyses

**Figure 4:**
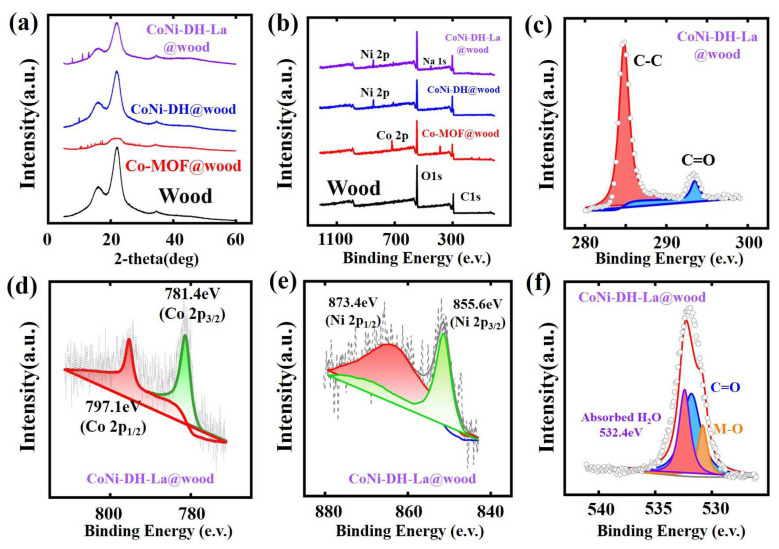
Chemical composition characterization of different wood samples: (**a**) XRD images of different wood samples; (**b**) XPS spectra of different wood specimens; (**c**–**f**) C1s, Co-2p, Ni-2p, and O1s in CoNi-DH-La@wood.

An X-ray diffraction (XRD) analysis and X-ray photoelectron spectroscopy (XPS) were applied to further analyze the surface chemical components and crystalline structures of different wood specimens. As shown in Figure 4a, the typical X-ray diffraction characteristic peaks of Co-MOF disappear in the obtained XRD curves for CoNi-DH@wood and CoNi-DH-La@wood owing to the complete transformation of the Co-MOF. In the XRD patterns ranging from 55° to 65°, one can see the characteristic peaks belonging to nickel and cobalt hydroxides for the CoNi-DH@wood and CoNi-DH-La@wood specimens. Notably, the XRD patterns of CoNi-DH-La@wood exhibit different characteristic peaks at 7.8°, 10.4°, and 13.0°, which belong to the (009), (0012), and (0015) reflections of the layered hydroxide components in the sodium laurate [35].

In addition, the XPS spectra allowed us to further analyze the chemical compositions of the different wood surfaces, and the XPS spectra are presented in Figure 4b. The XPS spectrum (Figure 4c,f) of the untreated wood shows only two elemental peaks, C1s and O1s. The XPS spectrum of the Co-MOF@Wood shows a strong Co-2p elemental peak compared to the untreated wood, which indicates that the Co-MOF was successfully covered on the surface of the wood. In contrast to the spectrum of the Co-MOF@wood, the disappearance of the N signal and the kick of the Ni signal can be seen in the CoNi-DH@wood. In addition, the superhydrophobic wood surface has a strong Na signal, which indicates the successful coverage of the sodium laurate on the wood surface. The high-resolution XPS for Co-2p in Figure 4d present two main deconvolution peaks at 781.4 eV and 797.1 eV. Figure 4e exhibits the two main deconvolution peaks of Ni-2p at 855.6 eV and 873.4 eV. Thus, the XRD and XPS findings indicate that the superhydrophobic coating was successfully applied on the wood surface.

### 3.3. Wettability and Self-Cleaning Performance of the Wood Samples

**Figure 5:**
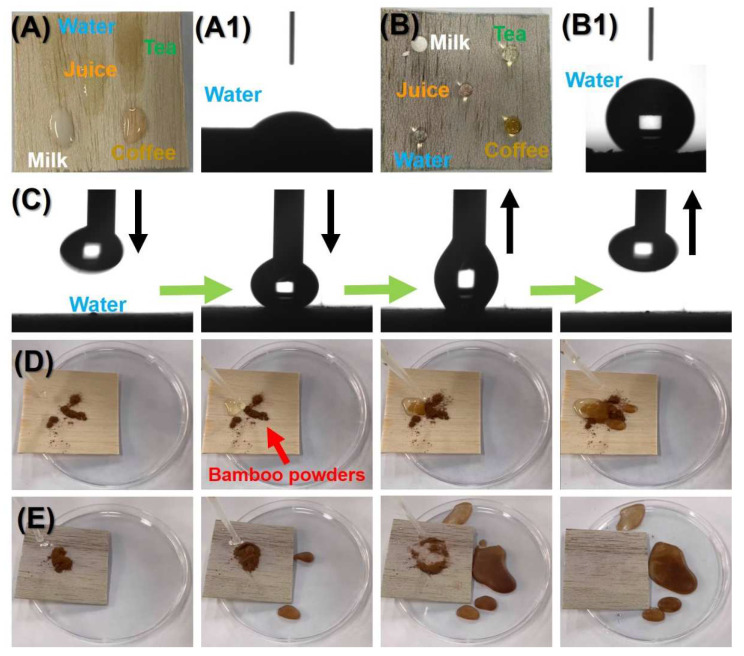
(**A**) Common household liquids on the untreated wood and the corresponding water contact angle (**A1**) of the balsa wood. (**B**) Common household liquids on the superhydrophobic wood and the corresponding water contact angle (**B1**) of the superhydrophobic wood. (**C**) Dynamic interaction between a water droplet and the superhydrophobic wood. (**D**) Self-cleaning performance of the untreated wood and (**E**) superhydrophobic wood.

As presented in Figure 5A,B, interestingly, for the CoNi-DH-La@wood, water-based droplets can maintain their initial shapes on the sample’s surface, such as from tea, milk, coffee, water, and apple juice, illustrating that the CoNi-DH-La@wood presents hydrophobicity. However, the untreated wood sample exhibits poor hydrophobic performance. The water contact angle of the CoNi-DH-La@wood is over 150°, illustrating the successful construction of a superhydrophobic coating on the balsa wood surface, endowing the wood surface with excellent superhydrophobic performance. In addition, the fabricated CoNi-DH-La@wood presented ultra-low water adhesion rates during the water contact angle test. For detail, the water droplets can be separated from the surface of the CoNi-DH-La@wood by lifting upwards.

In addition, the prepared superhydrophobic wood exhibits ultra-low water adhesion rates when the water droplet touches the surface (Figure 5C). The water droplet does not wet the surface and can be completely separated from the superhydrophobic wood surface by lifting upwards.

As shown in Figure 5D,E, the self-cleaning performance of the CoNi-DH-La@ wood was recorded using a camera with wood powder as the contaminant. At the same time, the superhydrophobic wood sample has excellent self-cleaning properties, as shown in Figure 5E, as water droplets can easily roll off the surface of the superhydrophobic wood and at the same time the wood powder adhering to the surface of the superhydrophobic wood are removed, leaving the surface of the superhydrophobic wood clean and tidy. The natural wood does not have superhydrophobic properties, so when water drops fall on the surface of the natural wood, the water droplets do not carry away the wood powder [36].

**Figure 6:**
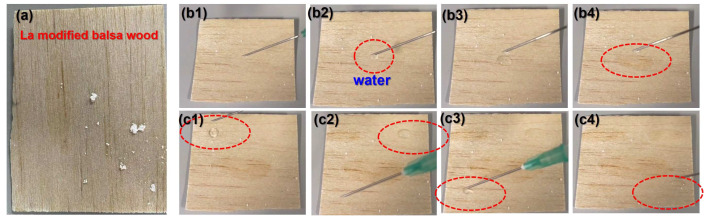
(**a**) La-modified balsa wood. (**b1**–**b4**, **c1**–**c4**) Hydrophilic test results on the wood surfaces.

**Figure 7:**
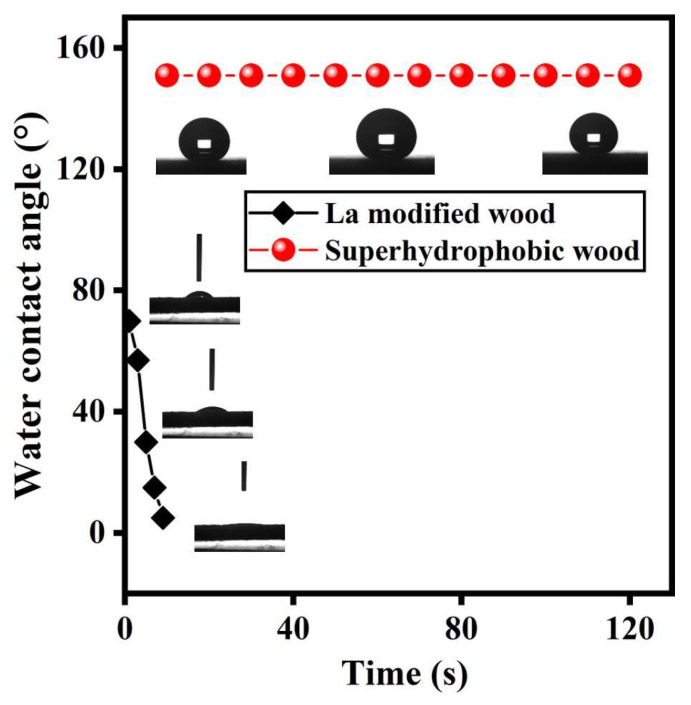
Water contact angles of different wood samples within 120 s.

Further, as can be seen from the SEM results in Figure 6a, the surface of the original wood sample is rough, and we applied sodium laurate directly to the surface of the original wood, corresponding to the wood sample shown in Figure 6a. It is not difficult to see that the surface of the original wood sample is whitish due to the direct application of sodium laurate on the surface. When we dropped a water droplet from the syringe on the surface of the wood modified with sodium laurate, it was not difficult to see that the water droplet disappeared quickly and completely within 3 s (Figure 6b1–b4), which demonstrated that the surface of the wood will not exhibit superhydrophobicity if the wood is modified with sodium laurate only. In addition, the surrounding area of the wood did not exhibit superhydrophobicity (Figure 6c1–c4). It can be seen in Figure 7 that the La-modified wood exhibited poor superhydrophobic properties and the water contact angle of the La-modified wood quickly decreased from 72.7° to 5° in 5 s. However, the water contact angle of the CoNi-DH-La@wood remained over 150° for 120 s, illustrating that the CoNi-DH-La@wood exhibited excellent superhydrophobic performance.

### 3.4. Durability and Stability of the Wood Samples

**Figure 8:**
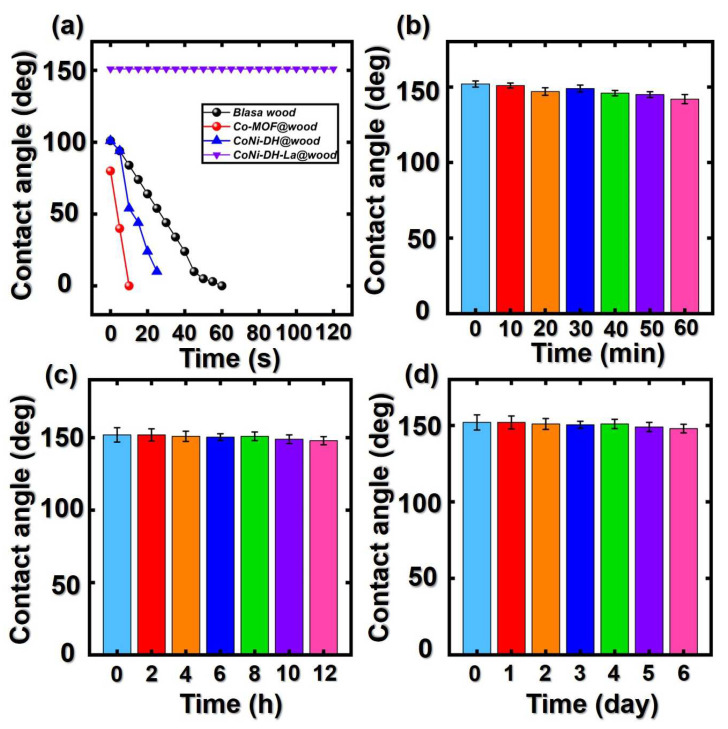
(**a**) Water contact angles of different wood samples within 120 s. (**b**) Water contact angle of CoNi-DH-La@wood after ultrasonic washing in water. (**c**) Water contact angle of CoNi-DH-La@wood after exposure to UV radiation. (**d**) Water contact angle of CoNi-DH-La@wood after exposure in an outdoor environment.

The water contact angle, chemical stability, durability, and environmental stability of the superhydrophobic layers on the CoNi-DH-La@wood were also analyzed in this work and the corresponding data are presented in Figure 8. As presented in Figure 8a, the water contact angle of CoNi-DH-La@wood remained over 150° for 120 s, illustrating that the CoNi-DH-La@wood exhibited excellent superhydrophobic performance. The water contact angle of the CoNi-DH-La@wood after ultrasonic washing in water, exposure in UV radiation, and exposure in an outdoor environment are presented in Figure 8b,c. Thus, the ultrasonic washing test was applied to investigate the stability of the superhydrophobic layers on the wood surface, and the water contact angle presented an downward tendency after 60 min of ultrasonic washing. Fortunately, the water contact angle of the washed CoNi-DH-La@wood still remained above 145°. At the same time, the superhydrophobic layer maintained its excellent superhydrophobicity after 6 days of outdoor testing and 12 h of UV radiation. All of the test results demonstrate that the superhydrophobic layer on the CoNi-DH-La@wood possessed excellent environmental stability.

**Figure 9:**
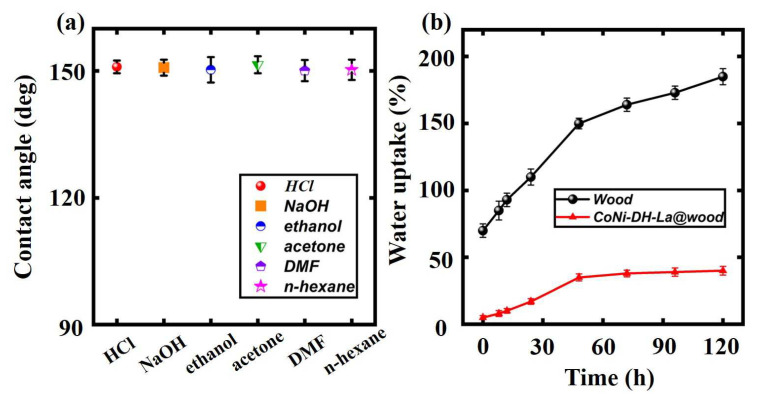
Chemical stability of the superhydrophobic layer: (**a**) water contact angles for the CoNi-DH-La@wood specimen soaked in different chemical solutions; (**b**) water uptake rates of the untreated wood sample and CoNi-DH-La@wood specimen.

The water contact angle of the superhydrophobic wood remained above 150° after 48 h of immersion in organic solutions with different PH values, such as HCl, NaOH, ethanol, acetone DMF, and n-hexane. This shows that the superhydrophobic wood can maintain its excellent superhydrophobic properties in acidic, alkaline, and organic solutions. In addition, the water permeability of the CoNi-DH-La coatings was evaluated based on the water uptake, as presented in Figure 9. For detail, the untreated balsa wood and CoNi-DH-La@wood samples were both immersed in water at room temperature, the wood samples weights were measured using a weighing balance every 24 h, and the data were recorded. The equation used to calculated the water uptake can be seen in the literature. In Figure 9b, the water uptake rates of both the untreated wood and CoNi-DH-La@wood increased over time. However, the water absorption rate of the untreated wood was relative quick, while the water absorption rate of the CoNi-DH-La@wood was slower. After 120 h of the water uptake experiment, approximately 180% of the water was absorbed by the untreated wood, while the CoNi-DH-La@wood absorbed approximately 40%. The water uptake test results illustrate that the superhydrophobic layer on the balsa wood surface positively impeded the moisture content from permeating the wood’s interior [37].

### 3.5. Mildew Resistance Performance of the Wood Samples

**Figure 10:**
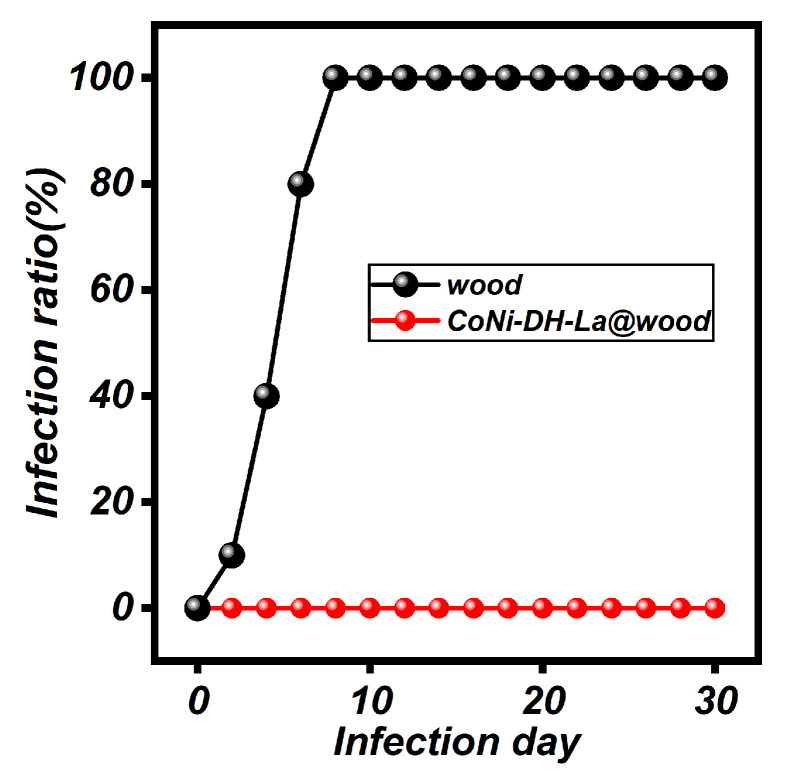
Infection rates of different wood samples in one month.

**Figure 11:**
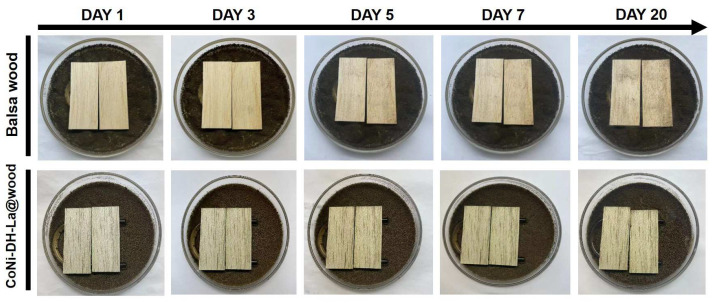
Physical photographs of wood samples during the anti-mildew test process over 30 days.

When the wood-based engineered products were applied for outdoor applications, the wood and wood-based engineered products were easily affected by mildew in this environment [38]. Thus, we investigated the anti-mildew performance of the fabricated superhydrophobic wood samples, and the related data are presented in Figure 10 and Figure 11. The anti-fungal performance of the fabricated CoNi-DH-La@wood was further analyzed. For detail, we recorded the development state and infection speed of the mildew on the wood surface within 30 days. The growing speed over 30 days and related experimental pictures are presented in Figure 10 and Figure 11. In the first 3 days, there was no observable fungal growth on the different wood specimens, while the wood samples absorbed moisture. As presented in Figure 10, the untreated balsa wood exhibited poor anti-mildew performance. For detail, the infection rate of the untreated wood sample quickly increased to 100% within the first 10 days, while the fabricated superhydrophobic wood was not infected after two days. After 8 days, the infection rate of the untreated wood quickly increased to 80%, and a lot of mold appeared on the wood surface, while the mildew area of the CoNi-DH-La@wood still equaled 0%. The enhancement of the anti-fungal ability of the CoNi-DH-La@wood can be attributed to the construction of the superhydrophobic layer on the wood’s surface [39]. On one hand, a superhydrophobic layer on a wood surface hinders water absorption and decreases water uptake, thereby further limiting mold growth on wood surface [40]. On the other hand, since the growth of mildew requires the participation of nutrients such as water, hemicellulose, cellulose, starch, and polysaccharides in the wood’s inner tissue, the growth of fungi is further inhibited by the successful construction of the superhydrophobic coating on the wood surface, which prevents the exchange of nutrients between the inside and outside environments. In addition, the reduced adhesion of the mildew to the wood surface can also positively affect the anti-mildew ability. The electrostatic effect caused by MOFs leads to the deformation of microbial cell walls, whereby they enter and react with the DNA, proteins, and nucleic acids in the microbial cells, inhibiting bacterial reproduction and growth. Thus, the CoNi-DH-La@wood exhibited excellent anti-mildew properties in comparison to the untreated wood specimens. Table 1 presents a comparison of anti-mildew wood and bamboo samples produced using different methods.

## 4. Conclusions

In recent years, as a class of popular crystalline materials, metal–organic frameworks (MOFs) have been widely applied in electrochemistry, adsorption, anti-mildew, and other applications. In this work, the in situ growth of metal–organic frameworks on wood surfaces resulted in the construction of micro- and nanohierarchical composite structures, while the modification of low surface energy substances using sodium laurate formed a superhydrophobic surface. The water contact angle of the CoNi-DH-La@wood reached up to 151° and remained virtually unchanged within a brief period. The CoNi-DH-La@wood exhibited excellent self-cleaning performance, as water droplets on the surface of the wood sample rolled off, taking away surface contaminants and keeping the surface dry and clean. In addition, the fabricated superhydrophobic balsa wood also exhibited excellent chemical and environment stability. The CoNi-DH-La@wood showed excellent anti-mold properties because of the presence of the superhydrophobic coating. After co-cultivation with *Aspergillus niger* for 30 days, the infection rates remained as low as 0%. In summary, the CoNi-DH-La@wood has excellent superhydrophilicity and anti-mildew properties, ensuring that it can be adapted to a wide range of complex locations.

## Figures and Tables

**Table 1:** Comparison of anti-mildew wood and bamboo samples produced using different methods.

Samples	Methods	Infection Ratio (%)	Ref.
Bamboo	Mesoporous aluminosilicate	75	[37]
Bamboo	Annealing processes	10	[38]
Wood	In situ growth of MOFs	0	This work

## Data Availability

Data are contained within the article.
